# Neem (*Azadirachta indica* A. Juss) Oil to Tackle Enteropathogenic *Escherichia coli*

**DOI:** 10.1155/2015/343610

**Published:** 2015-05-03

**Authors:** Paola Del Serrone, Chiara Toniolo, Marcello Nicoletti

**Affiliations:** ^1^Agricultural Research Council (CRA), Animal Production Research Centre (CRA PCM), Via Salaria 31 Km 26.700, 00015 Monterotondo, Italy; ^2^Department of Environmental Biology, University of Rome Sapienza, Piazzale Aldo Moro 5, 00185 Rome, Italy

## Abstract

Neem (*Azadirachta indica* A. Juss) oil (NO) was assayed against forty-eight isolates of *Escherichia coli* by standardised disc diffusion test and microdilution test. By molecular biology characterization, fourteen isolates resulted in diarrheagenic *E. coli* with sixteen primer pairs that specifically amplify unique sequences of virulence genes and of 16S rRNA. The NO showed biological activity against all isolates. The bacterial growth inhibition zone by disc diffusion method (100 *µ*L NO) ranged between 9.50 ± 0.70 and 30.00 ± 1.00 mm. The antibacterial activity was furthermore determined at lower NO concentrations (1 : 10–1 : 10,000). The percent of growth reduction ranged between 23.71 ± 1.00 and 99.70 ± 1.53. The highest bacterial growth reduction was 1 : 10 NO concentration with 50 *µ*L of bacterial suspension (ca. 1 × 10^6^ CFU/mL). There is significant difference between the antibacterial activities against pathogenic and nonpathogenic *E. coli*, as well as NO and ciprofloxacin activities. Viable cells after the different NO concentration treatments were checked by molecular biology assay using PMA dye. On the basis of the obtained results, NO counteracts *E. coli* and also influences the virulence of *E. coli* viable cells after NO treatment. The NO metabolomic composition was obtained using fingerprint HPTLC.

## 1. Introduction

Zoonotic food- and waterborne pathogens began resistant to antibiotics. It is now evident that antimicrobial resistance is an environmental problem. Detectable antibiotic residues are present in waste water from water treatment plants [1], and antibiotic-resistant bacteria can be isolated from ground water and soil [2, 3]. The cause of contamination may be* inter alia* the consequence of farming practices. Use of antibiotics, as growth promoters or for prophylaxis in farm animals, selects resistant strains of enterobacteria in gastrointestinal tract. These resistant strains have been also isolated from food and consequently this represents the main way to spread in the human gastrointestinal tract [4, 5]. The increasing incidence of foodborne diseases, coupled with the resultant social and economic implications, causes a constant striving to produce safer feed and food, as to develop new natural antimicrobial agents [6–8].

Meat contamination by pathogen bacteria may have great health consequence and high impact on consumers. The most known cases are related to HUS, hemolytic uremic syndrome, that was first recognized in 1982 in USA and Canada, with outbreaks associated with fast food restaurants. People experienced gastroenteritis with bloody diarrhoea, caused by the lining of their microbiota. In 1993, a multistate outbreak generated international interest in this disease, popularized by the name “hamburger disease.” Hamburger disease is based on association with the consumption of ground beef patties containing a pathogen* Escherichia coli*. This should not be confused with the related benign* E. coli* that is in the gut of every mammal. Many strains of* E. coli *are part of the nonpathogenic facultative flora of intestinal tract of humans and other mammals. However, some of them induce diseases of the gastrointestinal and urinary tracts or may affect the central nervous system [9].

On the basis of their pathogenetic mechanism, diarrheagenic* E. coli* strains include ETEC (enterotoxigenic* E. coli*), EIEC (enteroinvasive* E. coli*), EHEC (enterohemorrhagic* E. coli*), EPEC (enteropathogenic* E. coli*), EAEC (enteroaggregative* E. coli*), and DAEC (diffusely adherent* E. coli*). All of them cause serious economic losses in farm animal herds and are widespread in newborns [10] in developed and developing countries. There is a wide range of transmission possibilities of these pathogens, including direct contact, food, drinks, environment, and others [11]. Epidemiology and clinical symptoms of the disease are similar in various animal species but the majority of strains are species-specific. They differ particularly in the type of the expressed surface “adherence” antigen (adhesin or pilus). These microorganisms produce two main types of virulence factors, that is, adhesins and enterotoxins.

In this work, a collection of* E. coli* isolates was considered. They were different in geographical origin and source of isolation and showed different pathogenetic characteristics.

Consumers look for meat products of upgraded sensory quality and increased functional and nutritional properties, as well as guaranteed safety but yet less processing, and fewer additives or “technological” interventions. Plant derived extracts, or phytocomplex, as effective antimicrobial agents, offer an alternative to synthetic food additives.

Neem (*Azadirachta indica* A. Juss) is considered one of the most promising trees of the 21st century, for its great potential in pest management, environment protection, and medicine [12]. Neem oil (NO) is the most important derived product with a great market worldwide. It contains about one hundred biologically active compounds. The most famous constituents are several nortriterpenes, named limonoids, that is, azadirachtin, nimbin, nimbidin, and nimbolide, besides the predominant oily constituents. NO is the most commercially relevant product obtained from the seeds. The neem cake is remaining after the extraction process.

In our previous studies, the antibacterial activity of NO against* E. coli *was investigated [13, 14]. The antibacterial activity resulted highest in comparison to the neem cake extract against meat spoilage microorganisms. The aim of the present work was to evaluate NO capability to cope with plastic genome of* E. coli*.

## 2. Materials and Methods

### 2.1. Bacterial Strains and Growth Conditions

Forty-eight strains of* E. coli* were considered. Among them, seventeen (FLC isolates) were from microorganism's collection of the Fodder and Dairy Productions Research Centre of Lodi (CRA FLC) of CRA. They were isolated from milk and cheese. All strains were typed both phenotypically and genotypically. Phenotyping was made by the PhenePlate system for* E. coli* (PhP-EC, PhPPlate Microplate Techniques AB, Stockholm, Sweden) and genetic characterization by RAPD PCR technique [15].

Seventeen CVVI isolates were from microorganism's collection of the Institute of Veterinary Research and Development of Central Vietnam, Vietnam. These microorganisms were isolated from faeces of calves affected by diarrhoea.

Ten NL isolates were from microorganism's collection of the Department of Bacteriology of Wageningen UR Livestock Research, Wageningen University & Research Centre, Netherlands. They were isolated from faeces of piglets and calves. They are antigenically different and detectable using specific monoclonal antibodies towards different fimbria antigens by* in vitro* agglutination test [16]. Four reference strains were also considered (DSMZ and ATCC isolates). They were from international culture collections.

The cultivation/assay medium for* E. coli* was Minca + 1% Iso Vitalex Agar/Broth (Sifin, Berlin, Germany). Bacterial cultures for antibacterial testing were prepared by picking colony from 24-hour-old plates and suspending them in the broth medium (5 mL). Cultures were grown aerobically for 18 h at 37°C and 100 rpm. For antibacterial activity assay, 1 mL of each culture was diluted to 10^5^–10^6^ CFU/mL. The reference strains were grown on media and at the growth conditions as reported on products sheets.

### 2.2. Plant Extract

A commercial neem oil produced by Neem Italia (Manerba (BS), Italy) was used as test starting material (0.35% azadirachtin A). Total composition of the neem oil was checked by high performance thin layer chromatography [17].

Neem oil was diluted in Tween 80 (1 : 1 V/V; VWR, PBI International, MI, Italy) under agitation and sterilised by filtration through a 0.22 *μ*m Millipore express filter (Millex-GP, Bedford, OH, USA) before use in the experiment.

### 2.3. HPTLC Assay

#### 2.3.1. HPTLC System and Materials

The HPTLC system (CAMAG, Muttenz, Switzerland) consisted of (i) Linomat 5 sample applicator using 100 *μ*L syringes, connected to a nitrogen tank; (ii) ADC 2 chamber containing twin trough chamber 20 × 10 cm; (iii) immersion device III; (iv) TLC Plate Heater III; (v) TLC visualizer; (vi) TLC scanner 3 linked to winCATS software.

Solvents for extraction and HPLC grade solvents were purchased from Sigma-Aldrich and Carlo Erba (Milan, Italy). Glass plates 20 cm × 10 cm with glass-backed layers silica gel 60 (2 *μ*m thickness) were from Merck (Darmstadt, Germany). Before use, plates were prewashed with methanol and dried for 3 min at 100°C. Standards used in the HPTLC analysis were isolated from neem cake (i.e., salannin, azadirachtin A, and unsaturated and saturated lipids) in previous research [18] and data concerning isolation and identification are not reported, but they are available per request. Limonoids standards concentration was 2 mM.

### 2.4. Sample Application

Filtered solutions were applied with nitrogen flow. Operating conditions were syringe delivery speed, 10 s *μ*L^−1^ (100 nL s^−1^); injection volume, 2 *μ*L; band width, 6 mm; distance from bottom, 15 mm.

### 2.5. Development

**Figure 1:**
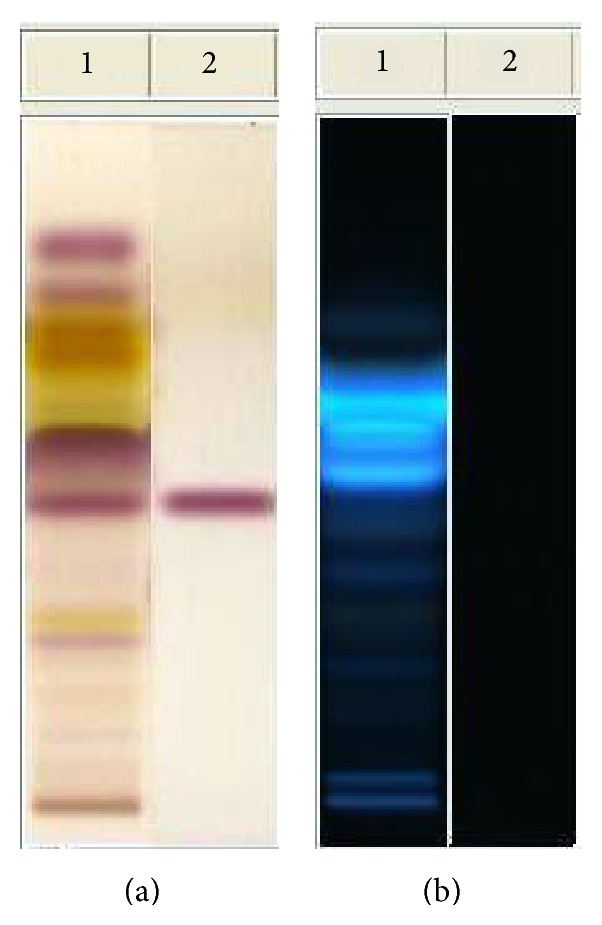
HPTLC analysis of neem oil EtOAc extract. Mobile phase: toluene : AcOEt 7 : 3 (v/v). Visualization: plate (a) (on the left) white light upper and lower; plate (b) (on the right) UV lamp at 366 nm. Derivatization: *p*-anisaldheyde. Track 1: neem oil; track 2: salannin.

The HPTLC plates were developed in toluene : AcOEt 7 : 3 (v/v) as mobile phase (Figure 1), in the automatic and reproducibly developing chamber ADC 2, saturated with the same mobile phase for 20 min at room temperature. The developing solvents (i.e., type of solvents and ratios) were carefully optimized before the analyses. The length of the chromatogram run was 80 mm from the point of application. The developed layers were allowed to dry in air for 5 min, derivatized with a selected solution, including *p*-anisaldheyde (1.5 mL *p*-anisaldheyde, 2.5 mL H_2_SO_4_, and 1 mL AcOH in 37 mL EtOH), dried in the open air, and then dipped into Macrogol reagent (1 g polyethylene glycol 400 in 20 mL of dichloromethane). Finally, the plates were warmed for 5 min at 120°C before inspection. All treated plates were inspected by a CAMAG TLC visualizer under a UV light at 254 or 366 nm or under reflectance and transmission white light (WRT), respectively, before and after derivatization.

### 2.6. Molecular Biology Characterization of the* E. coli* Isolates

Two primer pairs that amplify specific* E. coli* 16S rRNA sequences and fourteen primer pairs that specifically amplify target gene coding for virulence factors (adhesins and toxins) were employed to characterize the* E. coli* isolates considered in this study (Table 1). The PCR reaction mixtures and conditions are those as reported in the literature (Table 1).

The amplification products' sizes, coordinates, and accession numbers of each primer pair are shown in Table 2. Amplified products (7 *μ*L) were analyzed by electrophoresis in 2% or 3% agarose gels buffered in 0.5x TBE (TBE buffer: 90 mM tris(hydroxymethyl)aminomethane, 90 mM boric acid, and 3 mM ethylenediaminetetraacetate Na salt, pH 8.3, Sigma-Aldrich, Milano, Italy) against a 50 bp, 100 bp, and 1 Kb ladder used as size marker (Invitrogen, Milano, Italia) and visualized by UV light at 260 nm (Fotodine 3-3102 Celbio, Milano, Italy) after staining with ethidium bromide (3,8-diamino-5-ethyl-6-phenylphenanthridinium bromide, EtBr, Sigma-Aldrich, Milano, Italy).

### 2.7. Assessment of Antibacterial Activity

The antibacterial activity of the NO was assayed using standardized disc diffusion agar and microdilution methods. Disc diffusion method was carried out according to the standard method by Bauer et al. [19]. Bacteria cultures adjusted to 0.5 McFarland standard were used to lawn Muller Hinton agar plates evenly using a sterile swab. The agar plates were dried for 15 minutes. The discs impregnated with NO (100 *μ*L) were placed on the agar surface. Each test plate comprises three discs. The discs were placed equidistant to each other. Muller Hinton agar plates were set also up with positive control, which is the antibiotic ciprofloxacin (CFX) (100 *μ*L wt/v) (hydrochloride monohydrate 1 mg/mL, Bayer, Milano, Italy) and Tween 80 (TWN) (VWR International PBI Srl, Milano, Italy, 1 mg/mL) as negative control. The plates were then incubated at 37°C for 18 h. After the incubation, the plates and those considered as controls were examined for inhibition zone. The inhibition zones were then measured using calipers and were recorded. The plates were done in triplicate for each bacterial isolate and the experiment was performed twice. The results were recorded as mean ± S.D. of the duplicate experiment. Differences between means of data were compared by LSD calculated using the SAS.

The antibacterial activity of NO was also evaluated using microdilution method in conventional sterile polystyrene microplates (Corning, Euroclone SpA, Milan, Italy). Each well of the microplate was filled with 100 *μ*L of sterile suitable liquid media for each bacterial isolate considered, 50 *μ*L of inoculums and amounts of extract at lower concentrations (1 : 10–1 : 10,000) were added. Control treatment without NO was used in the experiment. The microplates were incubated at 37°C for 24 h. Bacterial growth was determined by OD reading at 630 nm/10 mm pathlength with an ELISA microplate reader (Dynatech ML-3000, Pina de Ebro, Spain). Bacterial cell concentration was transformed to cells/mL using the reference curve equation.

The reference curve was constructed by diluting at 1 : 100 each bacterial isolate. Counting the number of bacterial cells of an aliquot of this dilution was done using a Neubauer chamber (Celeromics, Vedano al Lambro, MI, Italy). Finally, cell concentrations were transformed to a percentage of bacterial inhibition. The percentage of bacterial growth reduction (GR%) was estimated using as reference the control treatment (*T* = without extract) as

(1) $$\begin{array}{c} \text{GR}\%=\frac{C-T}{C}\times 100. \end{array}$$

Three replicates were considered. The results were recorded as mean ± S.D. of the duplicate experiment. Differences between means of data were compared by least significant difference (LSD) calculated using the SAS.

## 3. Results and Discussion

### 3.1. Molecular Biology Characterization of the* E. coli* Isolates

The molecular biology characterization of the forty-eight* E. coli* isolates showed that fourteen isolates were diarrheagenic* E. coli*. They were ten* E. coli* isolated from feces of calves and piglets and four from calves collected, respectively, in Netherlands and Central Vietnam. Their virulence characteristics are reported in Table 3.

### 3.2. HPTLC Assay

The NO metabolomic fingerprint shows characteristic sequence of metabolites according to the polarity of constituents. The identification of the raw material was assured by the presence of salannin (Rf = 0.42), which is a typical maker of neem. In comparison with the spot of azadirachtin (Rf = 0.23), salannin appears as the main limonoid spot. Spots concerning lipids are present at Rf values at ca. 0.80, due to unsaturated fatty acids and fatty alcohols, and at Rf ca. 0.50, due to saturated and unsaturated triglycerides. The most interesting feature of the plate concerns the presence of compounds with high fluorescent reaction at between Rf 0.55 and 0.66, which are perfectly visible at 366 nm after derivatization with *p*-anisaldheyde. These spots can be attributed to compounds with high conjugated unsaturation in polycyclic aromatic structures, very different from those of the nortriterpenes limonoids, so far considered responsible for the activity. Therefore, more studies are necessary to decide about the importance of antibacterial activity of these substances in the phytocomplex.

### 3.3. NO Antibacterial Activity

The results obtained show that NO has a broad spectrum of antibacterial activities against the tested* E. coli* isolates. As shown in Table 4, the antibacterial activity was evaluated based on the diameters of clear inhibition zone surrounding the paper discs soaked with 100 *μ*L of neem oil. The NO average GIZ mm range from 9.50 ± 0.70 to 30.00 ± 1.00. The NO GIZ varies between enteropathogenic and nonenteropathogenic* E. coli* being, respectively, 24.33 ± 0.58–30.00 ± 1.00 and 9.50 ± 0.70–21.53 ± 1.53. It is significantly (*P* < 0.05) different with respect to the antibiotic activity. However, the* E. coli* isolate FLC1167 (from milk) resulted to be less susceptible and the* E. coli* isolate NLP097/F5 (from piglet feces) the most susceptible to NO treatment (100 *μ*L) among all tested bacteria using the disc diffusion method.

The CFX GIZ range is 0.00 ± 0.00–32.65 ± 75. The enteropathogenic* E. coli* resulted to be resistant or less susceptible to CFX than the nonenteropathogenic* E. coli*, showing a GIZ range of, respectively, 0.00 ± 0.00–18.24 ± 1.68 mm and 21.64 ± 0.94–32.65 ± 75 mm. The isolates CVVIK10B (from calve feces) and NLK99-3^*^ (from calve feces) both revealed resistance to CFX. No GIZ was detected in plates treated with negative controls (TWN and WTR).

As shown in Table 5, the percent bacterial GR revealed at 100 *μ*L, 10 *μ*L, 1 *μ*L, and 0.1 *μ*L NO concentrations was in the range 23.71 ± 1.00–99.70 ± 1.53; 21.61 ± 0.56–91.63 ± 0.08; 17.58 ± 1.33–69.57 ± 0.00; and 11.18 ± 0.89–67.58 ± 0.89.

There is a significant difference of antibacterial activity among the isolates and the NO concentrations tested (Table 5). The highest percent bacterial GRs were detected at 100 *μ*L NO and they concerned mainly enteropathogenic* E. coli* isolates. Amplicons of the expected sizes from virulence genes of enteropathogenic* E. coli* isolates were not detected when bacterial viable cells were checked in samples treated with 100 *μ*L and 10 *μ*L NO concentrations. On the contrary, amplicons of the expected size were revealed in the same samples using primer pair numbers 16 and 17, as reported in Table 1, that specifically amplify unique sequences of* E. coli* 16S rRNA.

The antibiotic activity of ciprofloxacin is to bind and inhibit bacterial topoisomerase types II and IV, thus being able to interfere with the bacterial processes of replication, transcription, and DNA repair.

An increasing ciprofloxacin resistance of* E. coli* isolates was reported [20] according several epidemiological studies.* E. coli*, the most commonly isolated bacterium in clinical samples from patients affected by different severity of diarrheal symptoms, shows high antibiotic resistance [21, 22]. Diarrheagenic* E. coli*, considered in the experiment, showed a resistance or less susceptibility to ciprofloxacin, in comparison with the other nonenteropathogenic isolates tested.

The viable cells of the fourteen diarrheagenic* E. coli* were checked after NO treatment with primer pairs listed in Table 1 and PMA dye. The dye propidium monoazide (PMA Biotium Inc., Hayward, CA, USA) is a photoreactive dye with high affinity for DNA. The dye intercalates into DNA and forms a covalent linkage upon exposure to intense visible light. It is cell membrane impermeable. When a sample comprising both live and dead bacteria is treated with PMA, only dead cells are susceptible to DNA modification due to their compromised cell membranes [23, 24]. Therefore, selective detection of the sole live cells is achieved.

The fourteen ciprofloxacin resistant/less susceptible diarrheagenic* E. coli* seem to lose their virulence after NO treatment, because amplicons were obtained only with the primer pairs numbers 16 and 17 (Table 1). This let us suppose that antibacterial activity acts on adhesion factor and membrane and its permeability with possible loss of extrachromosomal DNA.

## 4. Conclusions and Future Implications

Studies of new antimicrobials from plant derived extracts and agroindustrial byproducts as antimicrobials and preservatives are important issues in applied microbiology and biotechnology, for both implementing and improving effective alternative technologies to tackle antimicrobial resistance. The potential use of plant natural antimicrobials would require amendments of several different legal texts involving areas such as food additives, food packaging, and hygiene. Anyway, the applications could concern either the natural preservation in the food industries or an accessible and safe alternative to synthetic antimicrobial drugs.

## Figures and Tables

**Table 1:** Primer pairs used to specifically amplify target gene coding for virulence factors (1–9 = toxins; 9–15 fimbriae) of *E. coli *and 16S rRNA (16-17).

Target gene coding for virulence factors	Oligonucleotide sequences of primers	Reference
(1) LT	F 5′-ATT TAC GGC GTT ACT ATC CTC-3′ R 5′-TTT TGG TCT CGG TCA GAT ATG-3′	[25]

(2) Sta	F 5′-TCC GTG AAA CAA CAT GAC GG-3′ R 5′-ATA ACA TCC AGC ACA GGC AG-3′	[26]

(3) STb	F 5′-GCC TAT GCA TCT ACA CAA TC-3′ R 5′-TGA GAA ATG GAC AAT GTC CG-3′	[26]

(4) Stx1all	F 5′-CGC TGA ATG TCA TTC GCT CTG C-3′ R 5′-CGT GGT ATA GCT ACT GTC ACC-3′	[27]

(5) Stx2all	F 5′-CTT CGG TAT CCT ATT CCC GG-3′ R 5′-CTG CTG TGA CAG TGA CAA AAC GC-3′	[27]

(6) Stx2e	F 5-ATG AAG AAG ATG TTT ATA GCG-3′ R 5′-TCA GTT AAA CTT CAC CTG GGC-3′	[25]

(7) EAST1	F 5′-CCA TCA ACA CAG TAT ATC CGA-3′ R 5′-GGT CGC GAG TGA CGG CTT TGT-3′	[28]

(8) eae	F 5′-GGA ACG GCA GAG GTT AAT CTGCAG-3′ R 5′-GGC GCT CAT CAT AGT CTTTC-3′	[27]

(9) hlyA	F 5′-AGCTGCAAGTGCGGGTCTG-3′ R 5′-TACGGGTTATGCCTGCAAGTTCAC-3′	[29]

(10) F4 (K88)	F 5′-GCT GCA TCT GCT GCA TCT GGTATG G-3′ R 5′-CCA CTG AGT GCT GGTAGT TAC AGC C-3′	[30]

(11) F5 (K99)	F 5′-TGC GAC TAC CAA TGC TTC TG-3′ R 5′-TAT CCA CCA TTA GAC GGA GC-3′	[26]

(12) F6 (P987)	F 5′-TCT GCT CTT AAA GCT ACT GG-3′ R 5′-AAC TCC ACC GTT TGT ATC AG-3′	[25]

(13) F17	F 5′-GGG CTG ACA GAG GAG GTG GGGC-3′ R 5′-CCC GGC GAC AAC TTC ATCACC GG-3′	[30]

(14) F18	F 5′-GTG AAA AGA CTA GTG TTT ATT TC-3′ R 5′-CTT GTA AGT AAC CGC GTA AGC-3′	[31]

(15) F41	F 5′-GAG GGA CTT TCA TCT TTT AG-3′ R 5′-AGT CCA TTC CAT TTA TAG GC-3′	[26]

(16) E16SI	F 5′-CCCCCTGGACGAAGACTCAC-3′ R 5′-ACCGCTGGCAACAAAGGATA -3′	[29]

(17) E16SII	F 5′-AGAGTTTGATGGCTCAG-3′ R 5′-GGACTACCAGGGTATCTAAT-3′	[31]

**Table 2:** List of primer pairs' amplification products, coordinates, and accession numbers.

Target gene coding for virulence factors	Amplicon (bp)	Primer coordinates	Accession number
(1) LT	281	27–47, 287–307	S60731
(2) STa	244	267–286, 492–510	M58746
(3) STb	279	515–534, 773–793	AY028790
(4) Stx1all	302	113–134, 394–414	M17358
(5) Stx2all	516	50–69, 543–565	M59432
(6) Stx2e	264	1176–1196, 1419–1439	M36727
(7) EAST1	111	2–24, 94–114	S81691
(8) eae	775	1441–1460, 2193–2215	AF022236
(9) hylA	569	867-885, 1435–1412	X79839
(10) F4 (K88)	792	31–54, 798–822	M29374
(11) F5 (K99)	450	45–64, 475–494	M35282
(12) F6 (P987)	333	193–212, 506–525	M35257
(13) F17	411	289–310, 677–699	AF055313
(14) F18	510	1–23, 490–510	M61713
(15) F41	431	154–173, 565–584	X14354
(16) E16SI	401	1628-170, 2063–2082	AB035924
(17) E16SII	798	8-27, 798–805	J01859

**Table 3:** Molecular characterisation of enteropathogenic *E. coli* and reference strains considered in this study.

*E. coli* isolate collection's designation	Surface antigen	Toxins	Fimbriae
(1) CVVI K10B	nd	STb, LT, EAST1	F4
(2) CVVI KH10	nd	STa, STb	F18
(3) NLK99	O8K85K99	nr1	F5
(4) NLP987	O64 : K; 9877	STa+	F6
(5) CVVI E12b	nd	STa	F5, F41
(6) CVVI E10	nd	STa	F5, F41
(7) NLK99-1	O8 : K25 : K99	nr	F5
(8) NLK99-3	O101 : K28 : K99	nr	F5
(9) NLK99-5	O9 : K30 : K99	nr	F5
(10) NLK99-7	O101 : K32 : K99	nr	F5
(11) NLK99-9	O9 : K35 : K99	nr	F5
(12) NLK99-11	O9 : K37 : K99	nr	F5
(13) NLK99-15	O20 : K? : K99	nr	F5
(14) NLK99-19	O101 : K? : K99	nr	F5
(15) DSMZ8696	O55 : H6	nr	Nr
(16) DSMZ9025	—	—	—
(17) DSMZ10973	O6	nr	nr
(18) ATCC33559	—	—	—

CVVI: Central Vietnam Veterinary Institute; NL: Department of Bacteriology and Animal Science, University of Wageningen, Netherlands; DSMZ: Leibniz-Institut DSMZ—Deutsche Sammlung von Mikroorganismen und Zellkulturen GmbH; ATCC: American Type Culture Collection.

**Table 4:** Antibacterial activity of neem oil (NO) against forty-eight *Escherichia coli* isolates revealed as growth inhibition zone (mm).

*E. coli* isolates	Growth inhibition zone (mm)^*^			
	NSO (100 *µ*L )	TWN (100 *µ*L)	WTR (100 *µ*L)	CFX (100 *µ*L)
(1) FLC 1056	11.33 ± 0.58 b	—	—	30.41 ± 0.20 a
(2) FLC 1247	16.13 ± 1.15 b	—	—	30.52 ± 1.07 a
(3) FLC 1059	15.83 ± 1.13 b	—	—	29.62 ± 1.00 a
(4) FLC 1243	19.00 ± 1.00 b	—	—	31.53 ± 0.67 a
(5) FLC 1048	12.33 ± 0.58 b	—	—	29.42 ± 0.58 a
(6) FLC 1167	9.50 ± 0.70 b	—	—	30.61 ± 1.21 a
(7) FLC 1249	13.33 ± 0.58 b	—	—	29.61 ± 1.11 a
(8) FLC 1055	14.53 ± 1.25 b	—	—	31.75 ± 0.82 a
(9) FLC 1054	16.23 ± 1.18 b	—	—	31.41 ± 0.76 a
(10) FLC 1085	18.00 ± 1.00 b	—	—	30.53 ± 1.17 a
(11) FLC 1244	15.33 ± 0.48 b	—	—	28.86 ± 1.00 a
(12) FLC 1165	19.50 ± 0.70 b	—	—	31.33 ± 0.67 a
(13) FLC 1086	11.33 ± 0.58 b	—	—	29.82 ± 0.48 a
(14) FLC 1053	14.53 ± 1.15 b	—	—	32.65 ± 1.39 a
(15) FLC 1095	16.83 ± 1.18 b	—	—	29.05 ± 1.22 a
(16) FLC 1219	10.70 ± 1.00 b	—	—	32.75 ± 0.55 a
(17) FLC 1235	13.23 ± 0.88 b	—	—	30.15 ± 0.55 a
(18) DSM8696	13.50 ± 0.50 b	—	—	26.21 ± 1.00 a
(19) DSM9025	13.33 ± 0.58 b	—	—	21.64 ± 0.94 a
(20) DSM10973	13.53 ± 1.25 b	—	—	29.14 ± 1.75 a
(21) ATCC33559	13.83 ± 1.18 b	—	—	32.12 ± 1.09 a
(22) CVVI E210	13.00 ± 1.00 a	—	—	25.83 ± 1.65 a
(23) CVVI E173	12.23 ± 0.58 b	—	—	32.35 ± 1.49 a
(24) CVVI E12b	27.50 ± 0.50 b	—	—	11.25 ± 0.68 a
(25) CVVI E16	14.33 ± 0.88 b	—	—	27.54 ± 1.45 a
(26) CVVI E320	21.53 ± 1.35 b	—	—	28.75 ± 1.86 a
(27) CVVI E130	11.83 ± 1.78 b	—	—	29.64 ± 0.87 a
(28) CVVI E48	10.00 ± 1.40 a	—	—	29.31 ± 0.27 a
(29) CVVI KH10	26.33 ± 0.53 b	—	—	15.34 ± 0.66 a
(30) CVVI K10B	27.50 ± 0.56 b	—	—	0
(31) CVVI E298	11.33 ± 0.48 b	—	—	23.90 ± 1.69 a
(32) CVVI E273	13.53 ± 1.75 b	—	—	29.59 ± 1.77 b
(33) CVVI K436	13.14 ± 1.68 b	—	—	30.21 ± 1.38 a
(34) CVVI E98	11.00 ± 1.00 a	—	—	26.91 ± 1.56 a
(35) CVVI E77	14.33 ± 0.58 b	—	—	23.93 ± 0.59 a
(36) CVVI E148	13.50 ± 0.50 b	—	—	28.71 ± 0.87 a
(37) CVVI E10	24.33 ± 0.58 b	—	—	10.35 ± 1.11 a
(38) CVVI E215	16.53 ± 1.15 b	—	—	26.41 ± 1.40 a
(39) NLK99/F5	29.83 ± 1.18 b	—	—	16, 21 ± 0.89 a
(40) NLP987/F5	30.00 ± 1.00 a	—	—	13.21 ± 1.15 a
(41) NLK99-1^*^	21.73 ± 1.35 b	—	—	15.34 ± 1.37 a
(42) NLK99-3^*^	28.33 ± 1.50 b	—	—	0
(43) NLK99-5^*^	25.00 ± 1.10 a	—	—	9.54 ± 1.11 a
(44) NLK99-7^*^	26.33 ± 0.58 b	—	—	14,25 ± 1.11 a
(45) NLK99-9^*^	27.50 ± 0.50 b	—	—	11.26 ± 1.78 a
(46) NLK99-11^*^	29.53 ± 1.25 b	—	—	16.24 ± 1.68 a
(47) NLK99-15^*^	28.83 ± 1.38 b	—	—	16.35 ± 1.11 a
(48) NLK99-19^*^	25.00 ± 1.70 a	—	—	15.53 ± 0.84 a

Three paper discs per plate and three plates for each bacterium were considered. The experiment was repeated twice. Values are given as mean ± S.D. Values in a row followed by different lowercased letters are significantly different at *P* ≤ 0.05.

**Table 5:** Bacterial growth reduction (%) at 24 h in liquid medium with different concentrations of NSO, using as reference the control treatment (without NSO).

*E. coli* isolates	Percent growth reduction zone (%)			
	NSO (100 *µ*L)	NSO (10 *µ*L)	NSO (1 *µ*L)	NSO (0.1 *µ*L)
(1) FLC 1056	39.25 ± 1.43 c	35.61 ± 1.00 b	21.67 ± 1.33 a	11.31 ± 2.08 a
(2) FLC 1247	25.51 ± 1.15 c	24.70 ± 1.00 b	21.88 ± 1.33 a	11.86 ± 1.00 a
(3) FLC 1059	31.51 ± 1.15 c	25.70 ± 1.00 b	24.58 ± 1.33 a	14.86 ± 1.00 a
(4) FLC 1243	38.90 ± 1.00 d	28.79 ± 1.00 c	29.40 ± 0.00 b	16.68 ± 1.20 a
(5) FLC 1048	25.60 ± 1.53 d	23.73 ± 2.08 b	23.69 ± 2.00 b	12.83 ± 1.73 a
(6) FLC 1167	23.71 ± 1.00 b	21.61 ± 0.58 a	21.27 ± 0.00 a	11.18 ± 0.89 a
(7) FLC 1249	29.65 ± 1.53 b	28.61 ± 1.00 a	28.77 ± 1.33 a	18.51 ± 2.08 a
(8) FLC 1055	34.51 ± 1.15 c	29.70 ± 1.00 c	24.38 ± 1.33 a	14.86 ± 1.00 a
(9) FLC 1054	31.51 ± 1.15 d	29.70 ± 1.00 c	25. 78 ± 1.33 a	14.86 ± 1.00 a
(10) FLC 1085	39.90 ± 1.00 c	28.79 ± 1.00 b	28.10 ± 0.00 b	15.58 ± 1.20 a
(11) FLC 1244	35.70 ± 1.53 d	28.73 ± 2.08 b	28.59 ± 2.00 b	12.63 ± 1.73 a
(12) FLC 1165	39.71 ± 1.00 c	29.61 ± 0.58 c	29.17 ± 0.00 b	16.48 ± 0.89 a
(13) FLC 1086	39.55 ± 1.53 c	28.61 ± 1.00 c	27.87 ± 1.33 b	12.21 ± 2.08 a
(14) FLC 1053	34.51 ± 1.15 c	89.70 ± 1.00 c	27.58 ± 1.33 b	14.86 ± 1.00 a
(15) FLC 1095	31.51 ± 1.15 c	29.70 ± 1.00 b	25.18 ± 1.33 a	14.86 ± 1.00 a
(16) FLC 1219	38.95 ± 1.00 b	28.79 ± 1.00 b	28.10 ± 0.00 b	16.68 ± 1.20 a
(17) FLC 1235	36.70 ± 1.53 d	27.73 ± 2.08 c	27.79 ± 2.00 b	12.83 ± 1.73 a
(18) DSM8696	39.71 ± 1.00 c	29.61 ± 0.58 c	29.67 ± 0.00 b	17.58 ± 0.89 a
(19) DSM9025	39.65 ± 1.53 c	28.61 ± 1.00 c	26.17 ± 1.33 b	18.10 ± 2.08 a
(20) DSM10973	34.51 ± 1.15 c	29.70 ± 1.00 c	23.28 ± 1.33 b	14.86 ± 1.00 a
(21) ATCC33559	31.51 ± 1.15 d	29.70 ± 1.00 c	21.58 ± 1.33 b	14.86 ± 1.00 a
(22) CVVI E210	88.90 ± 1.00 c	88.79 ± 1.00 c	69.20 ± 0.00 b	14.78 ± 1.20 a
(23) CVVI E173	36.50 ± 1.53 d	27.73 ± 2.08 c	27.99 ± 2.00 b	22.83 ± 1.73 a
(24) CVVI E126	89.81 ± 1.00 c	89.61 ± 0.58 c	69.37 ± 0.00 b	44.58 ± 0.89 a
(25) CVVI E16	38.55 ± 1.53 c	28.61 ± 1.00 c	27.57 ± 1.33 b	11.51 ± 2.08 a
(26) CVVI E320	38.51 ± 1.15 c	29.70 ± 1.00 c	27.18 ± 1.33 b	14.86 ± 1.00 a
(27) CVVI E130	30.71 ± 1.15 c	29.70 ± 1.00 c	27.68 ± 1.33 a	14.46 ± 1.00 a
(28) CVVI E48	38.60 ± 1.00 c	28.79 ± 1.00 c	29.20 ± 0.00 a	16.68 ± 1.20 a
(29) CVVI KH10	99.60 ± 1.53 d	81.73 ± 2.08 c	68.39 ± 2.00 b	62.33 ± 1.73 a
(30) CVVI K10B	89.81 ± 1.00 c	89.61 ± 0.58 c	69.37 ± 0.00 b	60.58 ± 0.89 a
(31) CVVI E298	39.75 ± 1.53 c	28.61 ± 1.00 c	27.57 ± 1.33 b	20.11 ± 2.08 a
(32) CVVI E273	34.51 ± 1.15 c	29.70 ± 1.00 c	26.58 ± 1.33 b	22.86 ± 1.00 a
(33) CVVI K436	31.85 ± 1.15 d	29.70 ± 1.00 c	27.18 ± 1.33 b	15.86 ± 1.00 a
(34) CVVI E98	37.93 ± 1.00 c	28.79 ± 1.00 c	29.50 ± 0.00 b	16.68 ± 1.20 a
(35) CVVI E77	33.69 ± 1.53 d	21.73 ± 2.08 c	27.29 ± 2.00 b	19.83 ± 1.73 a
(36) CVVI E148	39.71 ± 1.00 c	29.61 ± 0.58 c	29.57 ± 0.00 b	22.58 ± 0.89 a
(37) CVVI E10	89.65 ± 1.53 c	88.61 ± 1.00 c	61.67 ± 1.33 b	50.79 ± 2.08 a
(38) CVVI E215	34.51 ± 1.15 c	29.70 ± 1.00 c	17.58 ± 1.33 b	21.86 ± 1.00 a
(39) NLK99/F5	91.51 ± 1.15 d	89.70 ± 1.00 c	67.68 ± 1.33 b	61.86 ± 1.00 a
(40) NL12B/F5	88.90 ± 1.00 c	88.79 ± 1.00 c	69.60 ± 0.00 b	63.68 ± 1.20 a
(41) NLK99-1^*^	97.70 ± 1.53 d	81.73 ± 2.08 c	68.69 ± 2.00 b	62.83 ± 1.73 a
(42) NLK99-3^*^	89.71 ± 1.00 c	79.61 ± 0.58 c	69.57 ± 0.00 b	67.58 ± 0.89 a
(43) NLK99-5^*^	89.65 ± 1.53 c	78.61 ± 1.00 c	67.67 ± 1.33 b	50.81 ± 2.08 a
(44) NLK99-7^*^	84.51 ± 1.15 c	79.70 ± 1.00 c	67.58 ± 1.33 b	44.86 ± 1.00 a
(45) NLK99-9^*^	91.51 ± 1.15 c	79.70 ± 1.00 c	67.28 ± 1.13 b	64.86 ± 1.00 a
(46) NLK99-11^*^	88.90 ± 1.00 c	78.79 ± 1.00 c	69.60 ± 0.00 b	66.68 ± 1.20 a
(47) NLK99-15^*^	99.70 ± 1.53 d	91.63 ± 0.28 c	68.69 ± 2.00 b	62.83 ± 1.73 a
(48) NLK99-19^*^	89.71 ± 1.00 c	79.61 ± 0.58 c	69.57 ± 0.00 b	67.58 ± 0.89 a

Three plates for each bacterium were considered. The experiment was repeated twice. Values are given as mean ± S.D. Values in a row followed by different lowercased letters are significantly different at *P* ≤ 0.05.

## Abbreviations

ATCC: American Type Culture Collection
CFX: Antibiotic: hydrochloride monohydrate
CRA: Agricultural Research Council, Italy
CVVI: Central Vietnam Veterinary Institute
DAEC: Diffusely adherent* E. coli*
DSMZ: Leibniz-Institut DSMZ—Deutsche Sammlung von Mikroorganismen und Zellkulturen GmbH
EAEC: Enteroaggregative* E. coli*
EHEC: Enterohemorrhagic* E. coli*
EIEC: Enteroinvasive* E. coli*
ELISA: Enzyme-linked immunosorbent assay
EPEC: Enteropathogenic* E. coli*
ETEC: Enterotoxigenic* E. coli*
GIZ: Growth inhibition zone
HPTLC: High performance thin layer chromatography
HUS: Hemolytic uremic syndrome
LSD: Least significant difference
NCE: Neem cake extract
NL: Department of Bacteriology and Animal Science, University of Wageningen, Netherlands
NO: Neem oil
OD: Optical density
PMA: Propidium monoazide
RAPD PCR: Random amplified polymorphic DNA polymerase chain reaction
Rf: Ratio between the migration distance of substance and the migration distance of solvent front
SAS: Statistical Analysis System (SAS Institute, Inc., Cary, NC, USA)
SD: Standard deviation
TWN: Tween 80
WTR: Sterile distilled water.
